# "I was tricked": understanding reasons for unintended pregnancy among sexually active adolescent girls

**DOI:** 10.1186/s12978-021-01078-y

**Published:** 2021-01-22

**Authors:** Anthony Idowu Ajayi, Sally Atieno Odunga, Clement Oduor, Ramatou Ouedraogo, Boniface Ayanbekongshie Ushie, Yohannes Dibaba Wado

**Affiliations:** Population Dynamics and Sexual and Reproductive Health, African Population and Health Research Center, Off Kirawa Road, Manga Close, Kenya

**Keywords:** Unintended pregnancy, Teenage pregnancy, Adolescent girls, Contraceptives

## Abstract

**Background:**

While the Kenya government is mobilizing high-level strategies to end adolescent pregnancy by 2030, a clear understanding of drivers of early unintended pregnancy in the country is a necessary precursor. In this study, we determine the prevalence, associated factors, and reasons for unintended pregnancy among sexually active adolescent girls (aged 15–19 in two Kenya counties with the highest rate of teenage pregnancy.

**Methods:**

We used the “In Their Hands” (ITH) program's baseline evaluation data. The study adopted a mixed-methods design with 1110 sexually active adolescent girls in the quantitative component and 19 girls who were either pregnant or nursing a child in the qualitative. We used adjusted and unadjusted logistic regression to model factors associated with unintended pregnancy among respondents. We used a thematic analysis of qualitative data to examine girls’ reasons for having unintended pregnancy.

**Results:**

Overall, 42% of respondents have had an unintended pregnancy; however, higher proportions were observed among girls who were 19 years (49.4%), double orphans (53.6%), never used contraceptive (49.9%), out-of-school (53.8%), and married (55.6%). After adjusting for relevant covariates, the odds of unintended pregnancy were higher among girls who resided in rural areas (AOR 1.64, 95% CI 1.22–2.20), had primary or no formal education (AOR 1.50 95% CI 1.11–2.02), and had never used contraceptive (AOR 1.69 95% CI 1.25–2.29) compared with their counterparts. Current school attendance was associated with a 66% reduction in the probability of having an unintended pregnancy. Participants of the qualitative study stated that the desire to maintain a relationship, poor contraceptive knowledge, misinformation about contraceptive side effects, and lack of trusted mentors were the main reasons for their unintended pregnancies.

**Conclusion:**

A massive burden of unintended pregnancy exists among sexually active adolescent girls in the study setting. Adolescent boys and girls need better access to sexuality education and contraceptives in the study setting to reduce early unintended pregnancy.

## Résumé

**Contexte:**

Alors que le gouvernement kenyan déploie des stratégies de haut niveau pour mettre fin aux grossesses chez les adolescentes d'ici 2030, il est nécessaire de bien cerner les facteurs qui favorisent les grossesses précoces non planifiées dans le pays. Dans cette étude, nous déterminons la prévalence, les facteurs associés et les causes des grossesses non planifiées chez les adolescentes sexuellement actives (âgées de 15 à 19 ans) dans deux comtés du Kenya où le taux de grossesse adolescente est le plus élevé.

**Méthodologie:**

Nous avons utilisé les données d'évaluation de base du programme « In Their Hands » (ITH). L'étude a adopté une approche mixte comprenant 1110 adolescentes sexuellement actives dans la composante quantitative et 19 adolescentes enceintes ou allaitantes dans la composante qualitative. Nous avons utilisé une régression logistique ajustée et non ajustée pour modéliser les facteurs associés à une grossesse non planifiée chez les répondantes. Pour ce qui est des données qualitatives, nous avons procédé à une analyse thématique afin d’examiner les facteurs expliquant les grossesses non planifiées chez les filles.

**Résultats:**

Globalement, 42 % des répondantes ont vécu une grossesse non planifiée ; toutefois, des proportions plus élevées ont été observées chez les filles âgées de 19 ans (49,4 %), celles doublement orphelines (53,6 %), celles qui n'ont jamais utilisé de moyens contraceptifs (49,9 %), celles non scolarisées (53,8 %) et chez celles qui étaient mariées (55,6 %). Après ajustement des covariables pertinentes, les probabilités de grossesse non planifiée étaient plus élevées chez les filles qui résidaient dans des zones rurales (AOR:1,64, IC 95%:1,22-2,20), avaient un niveau d'éducation primaire ou aucune éducation formelle (AOR:1,50 IC 95%:1,11-2,02), et n'avaient jamais utilisé de contraceptifs (AOR:1,69 IC 95%-1,25-2,29) par rapport à leurs congénères. La fréquentation scolaire actuelle était associée à une réduction de 66 % de la probabilité d'être confrontée à une grossesse non planifiée. Les participantes à l'étude qualitative ont déclaré que le désir d'entretenir une relation, une mauvaise connaissance des contraceptifs, de fausses informations sur les effets secondaires des contraceptifs et le manque de mentors de confiance étaient les principales raisons de leurs grossesses non planifiées.

**Conclusion:**

Un lourd fardeau lié aux grossesses non désirées existe parmi les adolescentes sexuellement actives de la zone étudiée. Les adolescents, garçons et filles, doivent avoir un meilleur accès à l'éducation sexuelle et aux contraceptifs dans la zone étudiée afin de réduire les grossesses non planifiées précoces.

## Introduction

Unintended pregnancy remains a significant public health concern worldwide, with a recent global estimate showing that 44% of all pregnancies between 2010 and 2014 were unplanned [1]. Notably, the rate of unintended pregnancy varies from country to country and even within countries [2]. Evidence indicates that unintended pregnancy is higher among unmarried women, adolescent girls, and women aged over 40 years than married women and those aged 20–39 years [2].

Addressing unintended pregnancy is critical given its adverse consequences, among which are intimate partner violence, depression, suicidal ideation, anxiety, stress, lesser relationship satisfaction and social support, and even death [3, 4]. Also, women with unintended pregnancies have lower odds of receiving prenatal care and higher odds of late initiation of antenatal care, which can adversely affect both mothers' and infants’ health and wellbeing [5–8]. Unintended pregnancy also increases the likelihood of abortion and unsafe abortion-related deaths [9], especially in sub-Saharan African settings, where most countries have restrictive abortion laws [10].

What is more, the consequences of unintended pregnancy on the physical, psychological, health, and socioeconomic wellbeing of adolescent girls are far more deleterious [11–13]. Teenage girls who experienced unintended pregnancy are stigmatized, more likely to drop out of school and get married or die from unsafe abortion [14–18]. In other words, the consequences of unintended pregnancy force adolescents girls to seek abortion from clandestine providers, lacking the minimum required medical standard, and under dangerous conditions, leading to avoidable deaths [19]. A study has shown that half of the abortion-related deaths in sub-Saharan Africa occur among adolescent girls and young adult women (aged 10–24) [18]. Unintended pregnancy could have long-term effects across adolescent girls' life course, especially on their social, economic, and educational outcomes. There is evidence that unintended adolescent childbearing harms literacy and numeracy, which are critical academic skills required not only for better income but that could lead to better health and more economic productivity [20]. Adolescent girls in sub-Saharan Africa (SSA) are particularly susceptible to unintended pregnancy due to gender inequality, poverty, sexual violence and coercion, national policies restricting access to contraception, child marriage, lack of access to sexuality education, and underinvestment in adolescent girls’ human capital [21–23].

While studies have focused on the prevalence and determinants of unintended pregnancy among women of reproductive age in general, studies focusing on the magnitude of unintended pregnancy among adolescent girls, especially in Kenya, are scarce. The few available studies from Kenya show that most pregnancies among adolescent girls are unplanned [24–26]. Estimating the prevalence of and factors associated with unintended pregnancy among adolescent girls as well as their perspectives on why they were vulnerable to unintended pregnancies is critical to developing policies and programs needed to tackle this problem. This study examines the prevalence of and factors associated with, as well as, reasons for unintended pregnancy among adolescent girls in two Kenya counties—Homabay and Narok—with the highest rate of teenage pregnancy.

Understanding the perspectives of adolescent mothers on reasons for their vulnerability to unintended pregnancy is essential, considering that Kenya records high prevalence of teenage pregnancy notwithstanding that the age of consent to sex is 18 years [27]. This study is also timely given the Kenya government’s International Conference on Population and Development (ICPD) 25 Nairobi Summit commitment to ending teenage pregnancy by 2030. Several key population stakeholders are already convening to identify and set critical priorities for ending teenage pregnancy in line with the national commitment. Efforts aimed at ending teenage pregnancy and unplanned pregnancy among adolescent girls must be evidence-informed. This study's findings could inform deliberations, policies, and programs to address unintended pregnancy among adolescent girls in Kenya.

## Methods

### Study area and study design

This study analyzed the baseline data of the evaluation of the “In Their Hands” (ITH) program. Three partners—Marie Stopes Kenya (MSK), Triggerise, and Well Told Story (WTS)—implemented the ITH program in 18 counties across Kenya to increase adolescents’ use of high-quality sexual and reproductive health services. The 18 counties were prioritized because they have high levels of teenage pregnancy, the highest unmet need for contraception among adolescents, and high rates of new sexually transmitted infections (STIs) and human immunodeficiency virus (HIV) infections [27]. As part of the project's evaluation, a baseline survey and qualitative study were undertaken in Homa Bay and Narok, out of the 18 counties involved in the main program because they have the highest rates of unintended pregnancy in Kenya. The study adopted a convergent mixed-methods cross-sectional design involving the concurrent collection of both qualitative and quantitative data between September and October 2018.

### Study population and sampling approach

The study involved adolescent girls aged 15–19 years who were residents and members of the sampled households in the selected counties for at least 6 months preceding the survey. A sample size of 1885 was calculated based on Cochran's formula for categorical data, but we successfully interviewed 1840 adolescent girls for the quantitative study. For this analysis, we included adolescent girls that have begun sex at the time of the survey—a total of 1110 respondents from both Counties. The sampling procedure involved a purposive selection of two counties with the highest prevalence of teenage pregnancy from the 18 counties where the ITH program is implemented. We then selected three sub-counties in each of the two counties. In each of the three sub-counties, we selected three wards based on the ITH affiliated health facilities' distribution. For each of the health facilities sampled for each ward, we identified seven and six catchment villages served by the health facility. Accordingly, we sampled 22 villages in Narok county and 24 villages in Homa Bay county. We conducted household listing in each of the villages and identified households with adolescent girls. We randomly selected only one adolescent girl for the interviews from each household with adolescent girls.

For the qualitative component, we purposively identified and interviewed 45 adolescent girls, 25 from Narok county and 20 from Homa Bay (varying by age, occupation, marital status, location, education level). Of the 45 adolescents interviewed, 19 (10 and nine from Narok and Homa Bay, respectively) met the criteria for inclusion in this paper—being pregnant or having had a baby at the time of the study. Community mobilizers identified adolescent girls in the study community, and we screened them to ensure that they met the inclusion criteria for the study. These included being a teenage girl aged 15–19 years, usual residence in any sampled communities two study counties. We defined “usual residence” as living in the household continuously for 6 months before the study.

### Instruments and procedures

Quantitative data were collected from a representative sample of adolescent girls living in urban and rural ITH areas to understand adolescents’ access to information, use of sexual and reproductive health (SRH) services, and SRH-related decision-making autonomy before implementing the intervention. We used an interviewer-administered structured questionnaire to collect the quantitative data using the ODK-based SurveyCTO platform, whereby interviewers used tablets in face-to-face interviews. We trained research assistants for five days on the study protocol, the study tools, and the informed consent process. Following training, we pre-tested the tools among adolescent girls in the Korogocho informal settlement in Nairobi county, which was not part of the study. We used the pilot test to check for consistency, sensitiveness, appropriateness, readability, and ease of understanding of the question; the feedback helped improve the quality of the tools. Piloting also helped in testing the quality of the instrument as programmed in SurveyCTO for the quantitative component. A team of 19 experienced research assistants (17 for quantitative and two for the qualitative) conducted the interviews.

The in-depth interviews with adolescent girls were conducted mostly in the Swahili language and partly in English, Dholuo, and Maasai languages, depending on the respondent's preference. We audio-recorded all interviews using digital recorders after obtaining permission from the interviewees. On average, each interview lasted an hour. We conducted all interviews in private spaces to guarantee confidentiality and allow respondents to freely voice their views and perspectives on contraceptives, sexual activities, and unplanned pregnancy, which are sensitive issues. We developed an interview guide for the study covering the mentioned topics.

### Ethical considerations

Amref Health Africa Ethics and Scientific Review Committee (AMREF-ESRC) approved our study protocol and materials (AMREF-ESRC P499/2018). Also, Kenya’s National Commission for Science, Technology and Innovation (NACOSTI) granted the research permit. Additional approvals were sought from local administrators and Ministries of Health and Education in the respective counties where the study was conducted. In the case of participants younger than 18, both parents/guardians and adolescents signed informed consent and assent forms, respectively, before interviews took place. However, married adolescents (considered emancipated) and those aged 18 years or older signed informed consent forms.

### Variables and measures

The primary outcome measure in this study was unintended pregnancy, which we define as not wanting to have or ambivalent about having a child or becoming pregnant at the time of index pregnancy. Responses were categorized into wanted, wanted to wait, wanted no more, and did not mind either way, but later re-categorized into the binary category of intended and unintended pregnancy for binary logistic regression analysis. The ambivalent cases were combined with the unintended category.

The covariates included in the study are individual-level factors (such as age, marital status, employment status, educational attainment, school attendance status, having ever used any contraceptives, and ownership of mobile phones), household /family-level factors (parents living status), and community-level factors (county of residence, and place of residence). These variables were included in the analysis based on existing literature on factors associated with unintended pregnancy [3, 28–31]. Age was measured as a continuous variable by asking respondents to state their age at their last birthday. We asked all respondents if they were currently married or not and if they own a mobile phone. Also, we measured parent living status by asking participants to indicate if any or both of their parents were alive or dead. Responses were classified as both parents alive, both parents dead, and only one parent alive. Place of residence was classified as rural or urban areas based on the population and amenities in the residence community. Employment status was measured by asking respondents if they have engaged in income-generating activity in the last six months preceding the interview. Binary choices of “yes” and “no” were provided. We also asked whether participants had dropped out or still in school. Lastly, we asked them to indicate the highest level of education attended and their highest grade completed. We classified education level into primary or less, and secondary or higher for the bivariate and multivariable analysis.

### Analytical approach

Based on evidence from previous studies that showed that estimating the prevalence of unintended pregnancy among adolescents, in general, may lead to biased estimates because a large proportion of them are not sexually active [32], we limited our analysis to 1110 adolescent girls who have initiated sex. Descriptive statistics-frequency counts and percentages, and inferential statistics-Pearson Chi-Square and Logistic Regression were performed. To examine the factors (individual, household, and community) associated with unintended pregnancy among adolescent girls, we fitted both adjusted and unadjusted regression models. The unadjusted model was the baseline model used to examine the relationship between each variable and unintended pregnancy. The adjusted model was used to investigate each individual level factor's overall effect after controlling for other relevant covariates (family and environmental level factors). The analysis was performed at a 95% confidence interval limit, and a *P*-value less than 0.05 was deemed to be statistically significant. We used Stata’s survey design data analysis feature to account for the complex sampling employed while also adding sampling weights.

All in-depth interviews were transcribed and translated into English. To ensure the translation's accuracy, we used two translators who understood the communities’ local languages and the English language. The transcripts were then verified against the related audios by two members of the study team, who are fluent in the Swahili language and English Language. The qualitative data coding was performed, using NVIVO, by two of the experienced qualitative researchers in qualitative methods. Emerging codes were compared and discussed among the research team, and the consensus was reached on the codes to include in the study. We used the thematic approach in developing the codebook and coding scheme and in the analysis.

## Results

Table 1 summarizes the demographic characteristics of respondents. The average age of study participants was 17.65?±?1.4 years. Almost all respondents were Christians (98.2%); the majority were single (65.3%) and out-of-school (60.0%). A significant proportion were half-orphans (38.1%); about one in two already had a child or were pregnant (53.9%), half of them owned a mobile phone (51.2%), and 50.8% had secondary education or higher. Ever-use of any contraceptive method for pregnancy prevention was 46.4%.

**Table 1:** Demographic characteristics of respondents

Variables	Frequency	Percentage
County		
Narok	451	40.6
Homa Bay	659	59.4
Place of residence		
Rural	592	53.3
Urban	518	46.7
Age (Years)		
15	129	11.6
16	126	11.4
17	141	12.7
18	323	29.1
19	391	35.2
Educational attainment		
Primary education or lower	546	49.2
Secondary education or higher	564	50.8
Current school attendance		
Still in school	444	40.0
Out of school	666	60.0
Religion		
Christian-Catholic	224	20.2
Christian-protestant and Pentecostal	866	78.0
Others (Islam, traditional or no-religion	20	1.8
Orphanhood status		
Both parents alive	684	61.6
Double-orphaned	110	9.9
Half-orphaned	316	28.5
Employed in the last six months		
Yes	305	27.5
No	805	72.5
Marital status		
Married	385	34.7
Single	725	65.3
Own a mobile phone		
Yes	568	51.2
No	542	48.8
Ever been pregnant		
Has a child or pregnant	598	53.9
Never had a child or pregnant	512	46.1
Ever used any contraceptives method		
Yes	515	46.4
No	595	53.6

While overall, more than two-fifths of all respondents had ever had unintended pregnancy, but significant variations exist across socio-demographic characteristics. Respondents who ever had unintended pregnancy were markedly higher among the 19-year-olds (49.4%), double-orphans (53.6%), those with primary education or lower (45.2%), the married (55.6%), out of school (53.8%) and those who never used contraception (49.9%).

The baseline regression model (see Table 2) suggest that increasing age, being married, double orphanhood, having only primary education or lower, and never use of contraceptives were associated with higher odds of unintended pregnancy. However, being in school was associated with lower odds of unintended pregnancy.

**Table 2:** Multivariable analysis showing predictors of unintended pregnancy among sexually active adolescent girls in Kenya

Variables	Had an unintended pregnancy	Model 1 (Unadjusted Odds ratios)	Model 2 (Adjusted Odds ratios)
All	461 (41.5)		
County			
Narok	181 (40.1)	1	1
Homa bay	280 (42.5)	1.10 (0.86–1.41)	1.24 (0.94–1.62)
Place of residence			
Urban	219 (42.3)	1	1
Rural	242 (40.9)	0.94 (0.74–1.20)	1.64 (1.22–2.20)*
Age			
15 years or less	25 (19.4)	1	1
16 years	36 (28.6)	1.66 (0.93–2.98)	1.73 (0.94–3.21)
17 years	57 (40.4)	2.82 (1.63–4.90)***	2.66 (1.45–3.21)*
18 years	150 (46.4)	3.61 (2.21–5.88)***	3.20 (1.81–5.66)***
19 years	193 (49.4)	4.05 (2.51–6.55)***	3.36 (1.88–6.02)***
Educational attainment			
Secondary education or more	214 (37.9)	1	1
Primary education or less	247 (45.2)	1.35 (1.06–1.72)*	1.50 (1.11–2.02)*
Orphan-hood status			
Both parents alive	263 (38.5)	1	1
Both parents dead	59 (53.6)	1.85 (1.23–2.78)*	1.37 (0.87–2.15)
Only one parent alive	139 (44.0)	1.26 (0.96–1.65)	1.15 (0.85–1.55)
Employed in the last 6 months			
Yes	131 (43.0)	1	1
No	330 (41.0)	0.9 (0.71–1.20)	1.30 (0.96–1.76)
Marital status			
Not married	247 (34.1)	1	
Married	214 (55.6)	2.42 (1.88–3.12)***	0.92 (0.66–1.29)
Own a mobile phone			
Yes	249 (43.8)	1	1
No	212 (39.1)	0.82 (0.65–1.05)	1.06 (0.80–1.41)
School attendance status			
Out of school	358 (53.8)	1	
Still in school	103 (23.2)	0.26 (0.20–0.34)***	0.34 (0.24–0.48)***
Ever used any contraceptives methods			
Yes	164 (31.8)		
No	297 (49.9)	2.13 (1.67–2.73)***	1.69 (1.25–2.29)*

***P-values?<?0.001

*P-values less than 0.05

The adjusted model results show that increasing age, rural residence, having primary education or lower, and never use of contraceptives were associated with higher odds of unintended pregnancy. On the other hand, current school attendance was protective; the odds of having an unintended pregnancy were 66% lower among adolescents attending school than those not currently in school. Respondents in rural areas were 64% more likely to have an unintended pregnancy relative to those in urban areas. Also, 19-year-old sexually active adolescents were over three times more likely to have an unintended pregnancy compared to those aged 15 years. Moreover, respondents whose education is primary or lower (AOR 1.50, CI 1.11–2.02) had higher odds of having an unintended pregnancy than those with secondary school or higher. Respondents who had never used any contraceptive methods were about twice more likely to experience an unintended pregnancy compared to those who had ever used contraceptives.

### Reasons for unintended pregnancy

The background characteristics of the adolescents who participated in the in-depth interviews (IDIs) are presented in Table 3 below. Seven of the 19 participants were married. Notably, only one of the participants was engaged in gainful occupation (as a domestic worker), with married adolescents being housewives.

**Table 3:** Background characteristics of the interviewees

County	Age (Years)	Occupation	Marital status	Type of residence
Narok	16	Currently out of school (primary) but intends to go back	Single	Rural
	17	Left school at primary due to pregnancy, and she is not planning to go back to school	Single	Rural
	17	Housewife	Married	Urban
	18	Out of school / staying at home due to pregnancy	Single	Urban
	18	Out of school due to pregnancy	Single	Rural
	18	Left school in class 7 after father passed away	Married	Urban
	19	None	Single	Urban
	19	Housewife—never been to school	Married	Rural
	19	Student (form IV)—has been out of school but intends to go back	Single	Rural
Homa bay	15	Pupil (no child but 5 months pregnant)	Single	Urban
	16	Housewife (left school at class 8 due to pregnancy)	Married	Urban
	16	Left school in class 8	Single	Rural
	16	Student (Form I)	Single	Rural
	16	Student (class 8 leaver, planning to join secondary school next year)	Single	Rural
	17	Out of school but living at home	Single	Rural
	18	Housewife—left school in form one due to pregnancy	Married	Urban
	19	Housewife	Married	Urban
	19	Housewife	Married	Urban
	19	Domestic worker—left school in form II after getting pregnant	Single	Urban

Participants in the qualitative component were asked reasons why many adolescents experience an early and unintended pregnancy. The findings show that the desire to maintain a relationship, poor knowledge of contraceptive methods for preventing unintended pregnancy, misinformation about side effects of modern contraception, and lack of trusted mentors were the main reasons for early unintended pregnancy, according to the participants. These reasons are thematized and elaborated below:

### “I was tricked”

Participants attributed their vulnerability to unintended pregnancy to the tenuous nature of their relationship situation. Some blamed the allure and charm of the boys as captivating and difficult to resist. They indicated that boys do challenge them to prove their love by engaging in sex with them, and they comply in a bid to impress the boys and maintain their relationships. Only for the boys to get them pregnant and abandon or deny them. Here is a conversation with a 19-year-old girl from Narok that aptly buttresses this theme:

Interviewer: You have told me about pregnancies and such; what causes people to have early or unintended pregnancies?
Respondent: Just being tricked.
Interviewer: Tell me how.
Respondent: You know sometimes boys do challenge us, they tell you they love you, but after they impregnate you, they avoid you and tell you that they don’t know you.
Interviewer: Tell me what happened in your case.
Respondent: I was just tricked. (Rural Narok, 19 years old, single with a child)

The challenge that boys usually throw at girls is also a form of trickery in which boys play on the girls' emotion. Boys appeal to girls’ emotions by playing the trick of insisting that without the proof of love, demonstrated through sexual intercourse, there is no love, and the girls, in proving their love, they engage in sex. While engaging in sex leads to pregnancy, the main challenge is the lack of protected sex that reduces the risk of pregnancy through contraceptives.

### Lack of contraceptive information before initiation of sex

The lack of information on how to prevent pregnancies before sexual debut emerged from the data as one of the main reasons for unintended pregnancy among adolescents. Interviewees tended to blame their parents and society for failing to inform them about how to prevent pregnancy. To many of them, the information on ways of preventing unintended pregnancy came too little too late as they were already pregnant before learning about pregnancy prevention. When asked about reasons for her unintended pregnancy, a 19-year-old adolescent mother in Homa Bay county affirmed that she lacked contraceptive information and knowledge, as neither her teacher, parents, nor and one else ever taught her about the methods of preventing unintended pregnancy. The excerpts below depict the lack of knowledge and information succinctly:

Interviewer: What was the main reason for your pregnancy?
Respondent: I think it is because I did not know how to prevent pregnancy at the time.
Interviewer: Speaking about how to prevent pregnancy, what did your parents tell you about contraceptives?
Respondent: Nothing.
Interviewer: Is there anyone who taught you about that?
Respondent: No.
Interviewer: How about at school—did teachers teach you? Have you ever heard anyone talk about family planning methods such as the use of condoms or injections?
Respondent: I have only heard of the use of injection, and that was when I was already pregnant

For some others, information about contraceptives came too late as they were already pregnant. An 18-year girl from Narok County who dropped out of school due to pregnancy noted that she only learned about contraceptives after becoming pregnant. She discussed contraception with her sister: she “had not talked about it before I became pregnant, but after I conceived. That’s when she started talking to me about it”. She now knows about contraceptives and pregnancy prevention so that “when I give birth to this one” (the pregnancy she was carrying), she will not “conceive again” as “maybe I will go for family planning.” She expressed some concerns about information at her disposal where people who used had side-effects, but knows that they “can find the method that will suit me”.

### Misconception about modern contraceptive methods

Besides the lack of contraceptive information, there appears to be misinformation and outright misconceptions regarding contraceptives' side effects among teenagers. These misconceptions present barriers to contraceptive use among young girls as there seems to be genuine fear and concern about the potential link between contraceptive use and future infertility. While significant others have not invested enough time to inform teenagers about ways of preventing pregnancy, they appear to have encouraged them not to use modern contraception. Often they warn them against using contraceptives to avoid future infertility. The instruction is explicit in teenagers’ minds: “do not use modern contraception until you have a child. Failure to comply means they will not be fertile in the future”. The following response by a 16-year-old interviewee from Homa Bay county who got pregnant and dropped out of school before the age of 15 supports this point:

Yes, we were told, but we were told that if you want to – you have to have a child first because if you just get into freely [use of contraceptives], you may fail to get a child – it affects. As in, if you start using the protections before you give birth or have a child, it can be a problem

A 17-year-old participant from Narok County believed that it is a community norm for young girls not to use modern contraception. According to her, the belief that modern contraception causes infertility is entrenched in their community. She responded when asked about the use of contraceptives:

In Maasai [name of her community], girls are not taken to clinics for contraceptives. I don’t know why. We have never known to date. They believe that if you go for family planning, you will never have children at all.

When asked if she knows anyone who has ever experienced infertility as a result of contraceptive use or it is something that they just believe, she responded by stating that she has never seen or heard of anyone who became infertile as a result of contraceptive use and explained that people just have that belief in their community.

### Lack of trusted mentors

Lack of trusted people to counsel young girls confidentially on sexuality issues in general and pregnancy prevention, in particular, was also reported as one of the leading causes of early unintended pregnancies. The fear that their private information will be exposed and they will be ridiculed for engaging in sex prevented these girls from seeking counsel from older girls in the community. When asked why she did not consult older women for information on how to prevent pregnancy, she responded, stating: *I don’t have an older woman I can trust. If you tell them, they will know you are having sex, and everyone will soon know*. Girls generally responded that they did not consult anyone because of a lack of trust and the high possibility of breach of confidentiality. In many of these communities, sex is viewed as a realm requiring adult maturity, which adolescent girls lack. As such, teenage girls are not to engage in sex. This judgemental portrayal of adolescent sexuality makes it difficult for teenage girls to confide in older women in their community about sex.

## Discussion

In this study, we examined the prevalence of and associated factors for unintended pregnancy among sexually active adolescent girls in two Counties in Kenya. We also explored the perspectives of adolescents on reasons for their vulnerability to an unintended pregnancy. We found a high level of unintended pregnancy among sexually active adolescent girls in the study setting. The unintended pregnancy rate reported in this study is far higher than the rate found among the general population of women of reproductive in Kenya [26]. Overall, this finding suggests that efforts to prevent teenage pregnancy have to focus on preventing unintended pregnancy. Our analysis also shows that a higher proportion of older adolescents, adolescents with primary or lower education, adolescents not currently attending school, married adolescents, and those who never used contraceptives experienced an unintended pregnancy. Our findings are not surprising, given a high rate of early unintended pregnancy has been reported in previous studies [24, 26]. In the Kenyan context, unintended pregnancy is among the drivers of child marriage, unlike in other settings where child marriage drives teenage pregnancy [33].

The qualitative study results show that the desire to maintain a relationship, poor contraceptive knowledge, misinformation about contraceptive side effects, and lack of trusted mentors were the main reasons for unintended pregnancies among childbearing adolescents. From our data, it is clear that adolescent girls want to enter relationships and see sex as a means of maintaining relationships with their boyfriends. However, the lack of reliable contraceptive knowledge and trusted mentors to guide them on how to prevent pregnancy makes them susceptible to an unintended pregnancy. The role of a trusted mentor, with whom adolescents can share confidential information about relationships, sex, and pregnancy prevention, is critical to reducing early unintended pregnancy in Kenya and other SSA settings. Previous studies have highlighted the significance of mentors in reducing risky sexual behaviors among adolescents [34, 35].

One notable finding of this study is that sexually active adolescent girls in rural areas were more likely to experience an unintended pregnancy relative to those in urban areas. While several studies [28, 36, 37] did not find a significant difference between the place of residence and unintended pregnancy, our finding is consistent with a multi-country study that examined the influence of place of residence on unintended pregnancy in SSA [2]. Obviously, access to contraceptive information and services is more for urban relative to rural dwellers. Consequently, adolescent girls in rural areas are less knowledgeable about pregnancy prevention methods and face more challenges accessing contraceptives when they desire. With a lack of information comes limited autonomy and capacity among rural adolescents to negotiate protected sex compared to their urban counterparts. Previous studies have alluded to urban advantage in access to health care services in SSA [38, 39]. Thus, higher unintended pregnancy among adolescent girls residing in rural areas may reflect rural disadvantages in access to family planning services. It is also plausible that sexually active adolescent girls in rural areas are more likely than their urban counterparts to experience other risk factors like poverty and sexual violence, which could potentially increase their likelihood of unintended pregnancy. However, this assumption requires further investigation. This finding suggests the need for policymakers to prioritize adolescent girls in rural areas for targeted interventions.

Consistent with previous studies [24, 26], our study shows that unintended pregnancy increases with increasing age. Adolescent girls are more vulnerable to unintended pregnancy as they increase in age, with those aged 19 years being over three times more likely to have an unintended pregnancy than those aged 15 years. As adolescent girls age, they mature biologically, making them attractive to their male counterparts. As they get to age 18—the legal age of adulthood—they have more freedom and express this freedom by engaging in relationships with the opposite sex. Further, they become more susceptible to peer pressure to engage in sex, as evidence shows that the proportion of adolescent girls who had engaged in sex increases with increasing age [40, 41]. With the poor implementation status of comprehensive sexuality education or life skills variant in Kenya schools, and even though more adolescent girls engage in sex as their age increases, they remain unprepared for preventing unintended pregnancy.

Lack of contraceptive use is another factor associated with unintended pregnancy in this study. Our study shows that sexually active adolescent girls who had never used any contraceptive method were almost twice as likely to experience an unintended pregnancy than those who had ever used any contraceptive methods. This finding is consistent with a study that explored the contribution of the underuse of modern contraception to the incidence of unintended pregnancy among adolescent girls [42]. Many sexually active adolescent girls who want to prevent pregnancy are not using any contraceptive methods, and several of those who ever used any method rely on less effective methods [40–43].

Our study also reemphasizes the connection between education level and the risk of unintended pregnancy and shows that education is a protective factor. We found that adolescent girls who had primary education or lower had a higher likelihood of experiencing an unintended pregnancy than those with higher qualifications. Likewise, sexually active adolescent girls who were still in school had a lesser probability of unintended pregnancy than their out-of-school counterparts. In some instances, adolescent girls drop out of school due to unintended pregnancy, but some become susceptible by dropping out of school. Studies have shown that school dropout is among the consequences of teenage pregnancy [44, 45].

Our findings suggest the need to prioritize two main proven interventions as policymakers deliberate on effective strategies to end teenage pregnancy in Kenya. First, comprehensive sexuality education improves knowledge of STI/HIV, reduces unintended pregnancy and unsafe abortion, increases contraceptive use, delays sexual debut, reduces the number of sexual partners, and enhances female autonomy in deciding when, how, and with whom to have sex [46, 47]. Therefore, it is critical to prioritize comprehensive sexuality education to equip young girls and boys with knowledge of pregnancy prevention before they initiate sex. It is also important to complement the provision of comprehensive sexuality education with increased access to contraceptives to end early unintended pregnancy in Kenya. Structural barriers such as cultural norms and values about sex and legal restriction on access to contraceptives must be addressed to end early unintended pregnancy.

### Study strengths and limitations

Even though this study provides evidence of early unintended pregnancy among sexually active adolescent girls, it has some limitations. The cross-sectional nature of the study means that the association reported cannot be interpreted as causation. Our study did not include data on other behavioral factors, such as alcohol and drug use, which are relevant predictors of unintended pregnancy. Moreover, given that sex is a sensitive topic, prone to social desirability bias, we could not rule out under-reporting of sexual activity, thus, impacting our denominator for estimating the prevalence of unintended pregnancy. We mitigated this by conducting the interviews in private spaces to engender trust and confidence among respondents, thereby ensuring reporting accuracy. Despite the limitations, our mixed-methods design is a strength of this study and helped generate robust data to understand why sexually active adolescent girls are susceptible to unintended pregnancy.

## Conclusion

There is a high prevalence of unintended pregnancy among adolescent girls in the study areas. Lack of contraceptive knowledge, desire to maintain a relationship, lack of trusted mentors, and misinformation about contraceptive side effects were the main drivers of early unintended pregnancies among adolescent girls. These findings underscore the need for interventions that focus on improving sexual and reproductive health knowledge of adolescents and expand access to contraception among adolescents and young people.

## Data Availability

The data will be made available by the corresponding author upon request.

## Abbreviations

HIV: Human immunodeficiency virus
ICPD: International Conference on Population and Development
IDI: In-depth interviews
ITH: In their hands
SSA: Sub-Saharan Africa
STI: Sexually transmitted infection
